# Supplementary material for: Functional role of the Frizzled linker domain in the Wnt signaling pathway
Source: Commun Biol. 2022 May 5;5:421. doi: 10.1038/s42003-022-03370-4 (PMC9072438; doi:10.1038/s42003-022-03370-4)
Supplement: Supplementary file 2 — Supplementary information [file 42003_2022_3370_MOESM2_ESM.pdf]

Functional role of the Frizzled linker domain in the Wnt signaling pathway

Supplementary Figures 1–23

Supplementary Table 1

Supplementary Figure 1

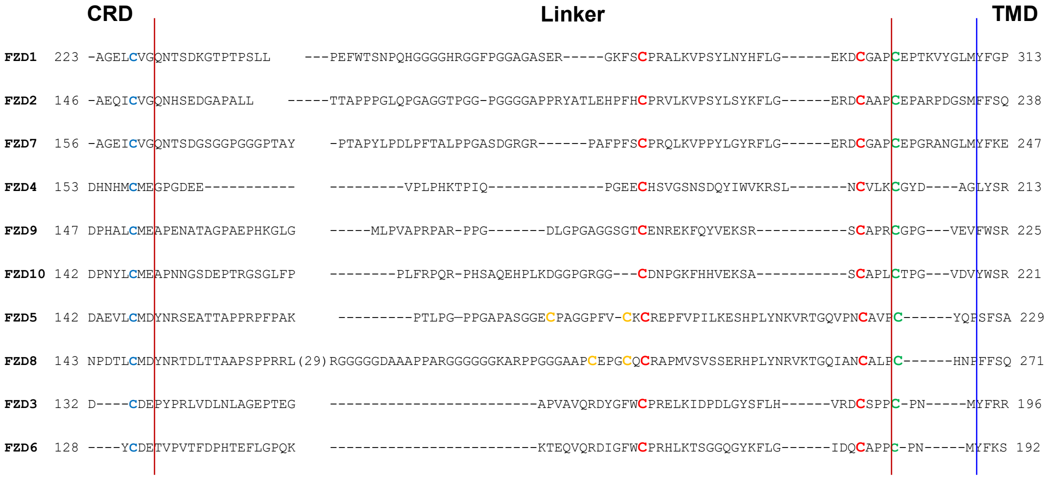

Supplementary Figure 1 Sequence alignment of linker domains in the FZD subtypes. Conserved disulfide bond forming cysteine residues in the linker domain are colored red. Extra cysteines in FZD5 and FZD8 are shown in orange and the conserved cysteine in CRD are in blue. Another conserved cysteine (green) that follows the red pair of cysteine is known to form a disulfide bond with cysteine in ECL2. We swapped FZD linkers within the boundaries drawn using red lines. The omitted 29 amino acid sequence of FZD8 is displayed as (29) for simplicity.

Supplementary Figure 2

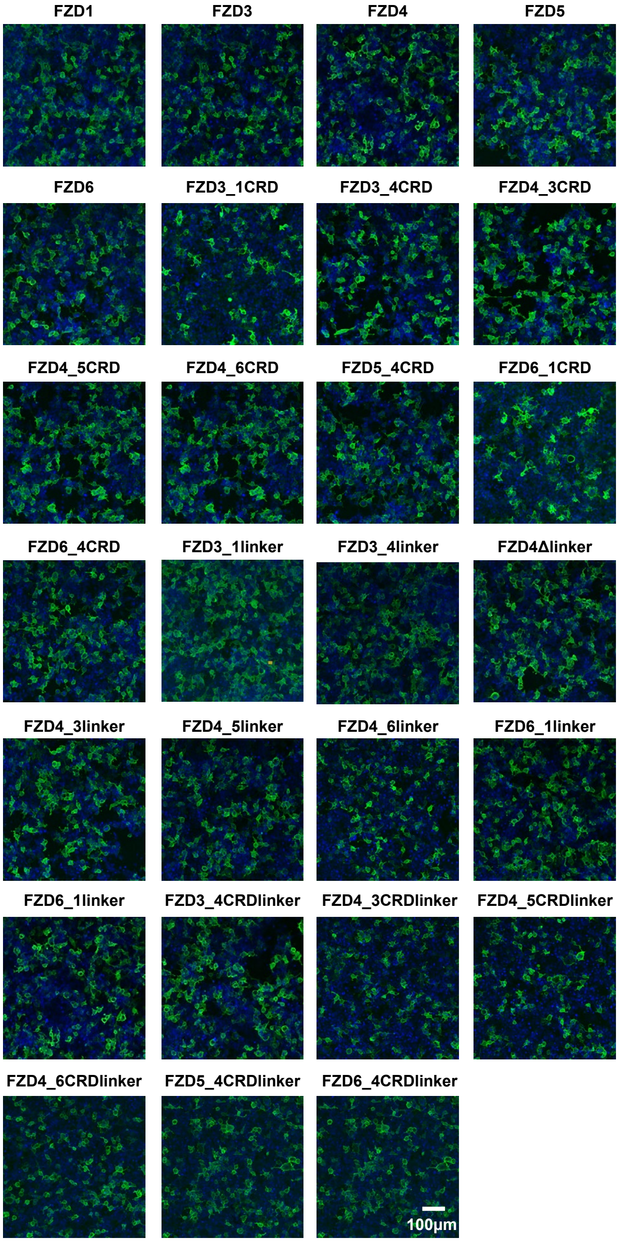

Supplementary Figure 2. Immunofluorescence assay of cells overexpressing various FZD constructs. Expressed FZDs were detected with anti-FLAG antibody and Alexa488 conjugated secondary antibody (green) and nuclei were stained with Hoeschst 33432 (blue). Scale bar indicates 100 μm.

Supplementary Figure 3

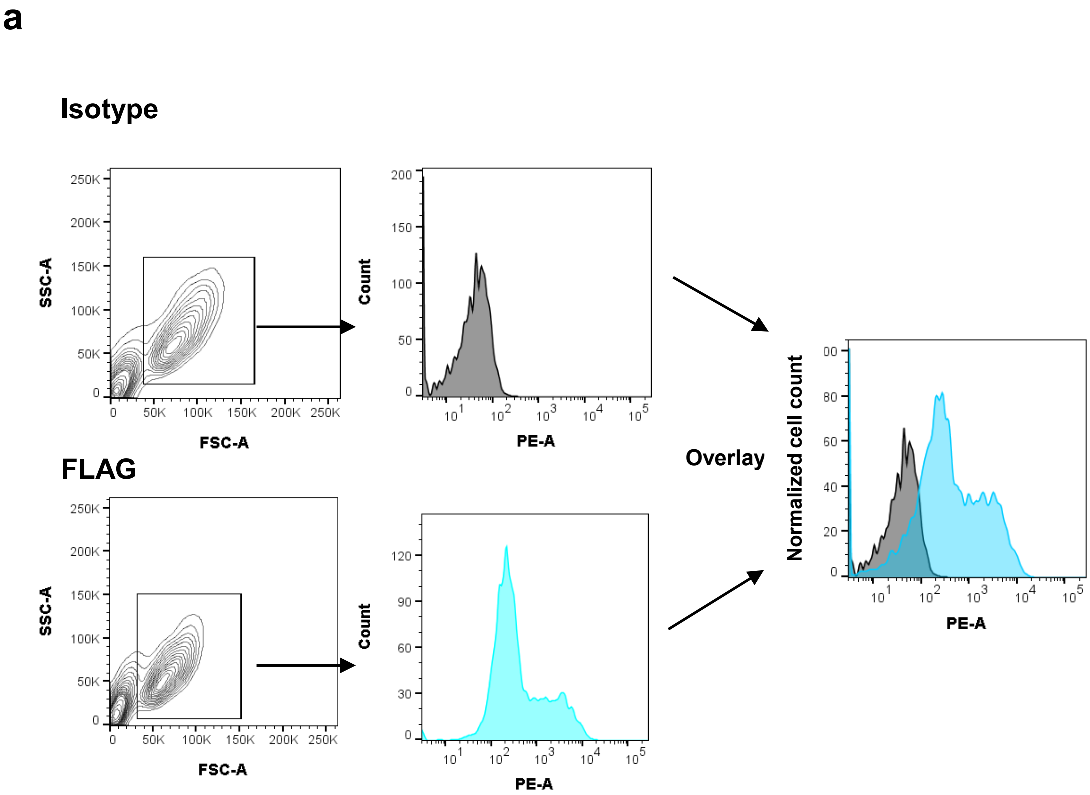

Supplementary Figure 3

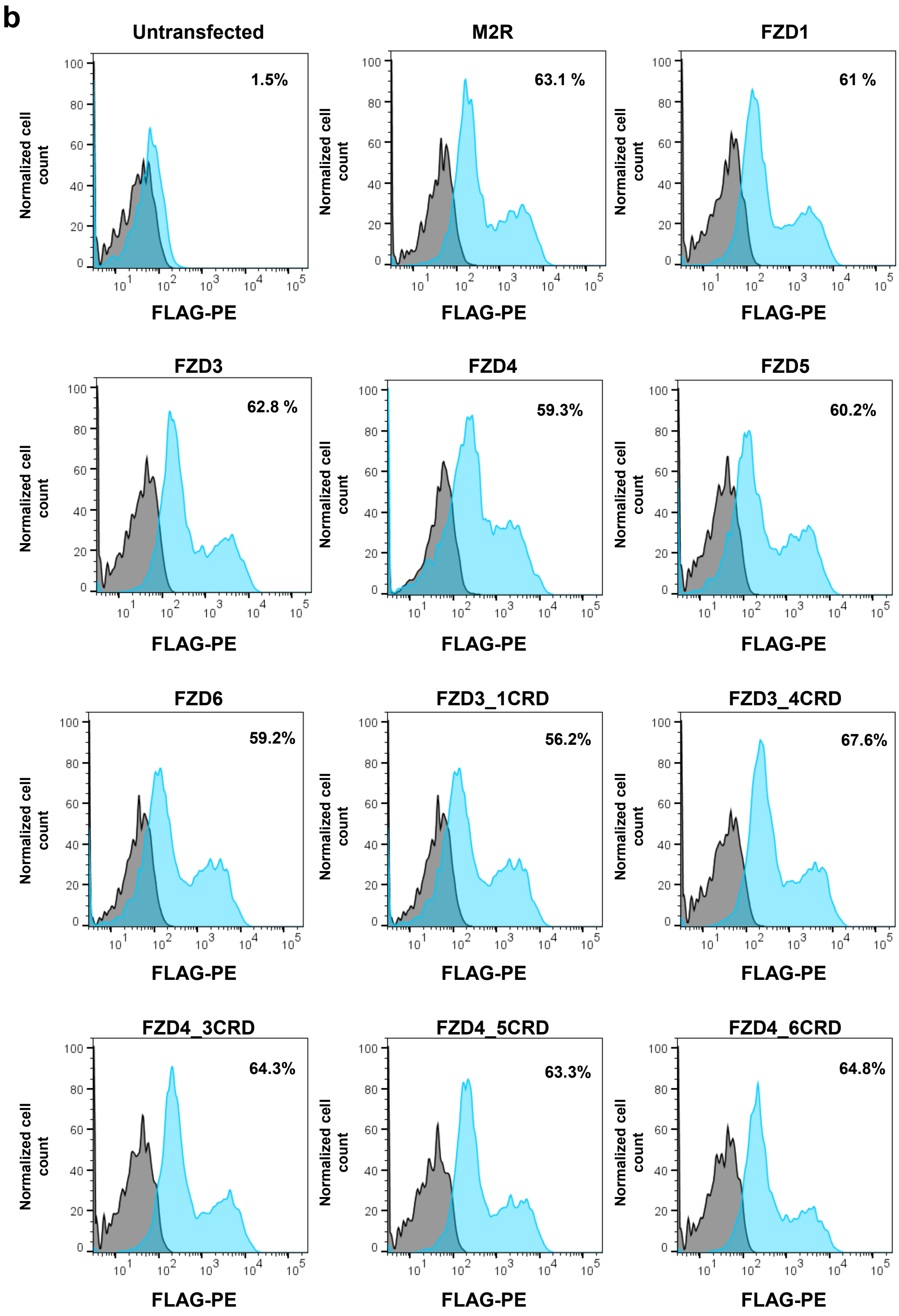

Supplementary Figure 3

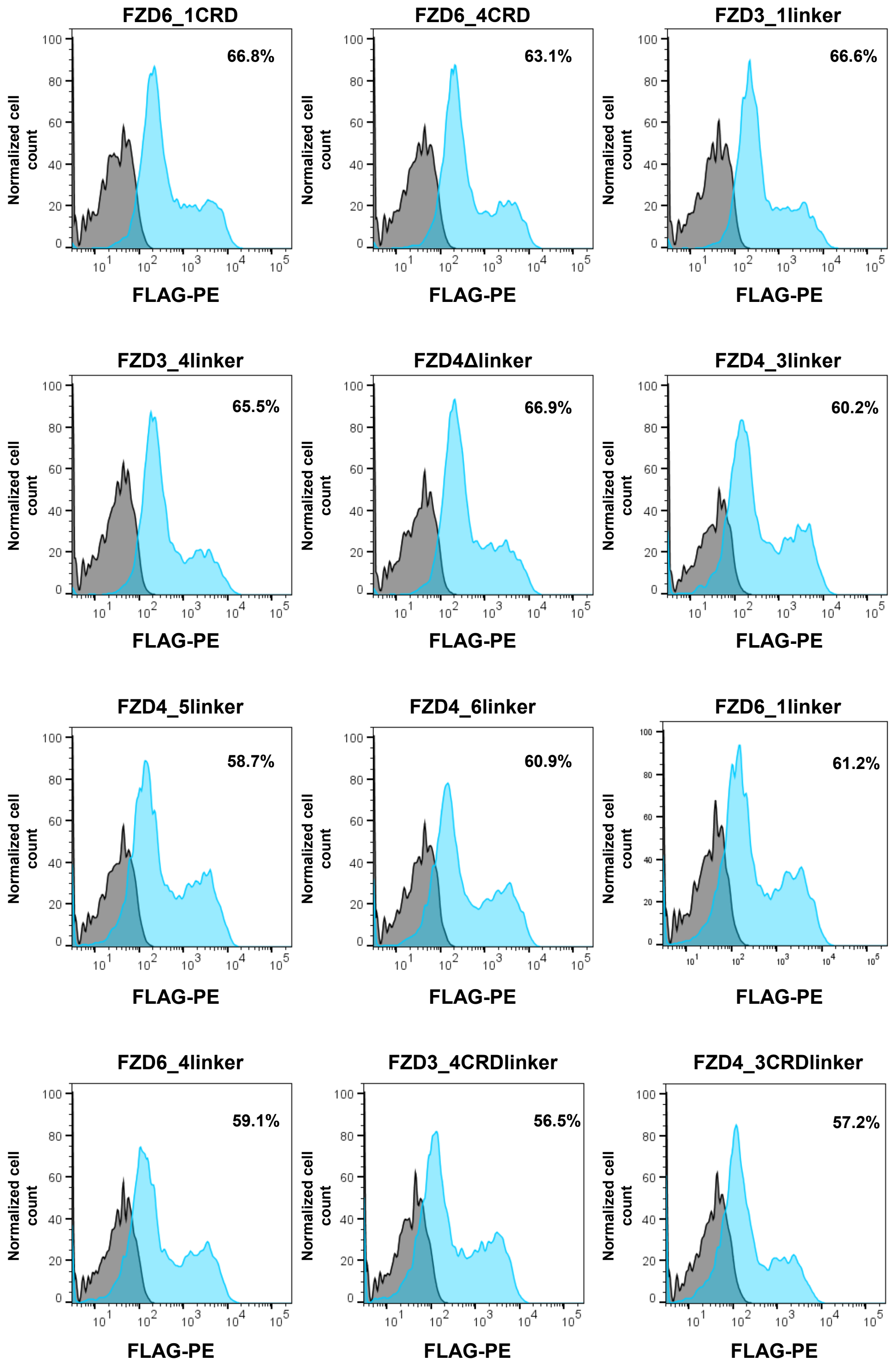

Supplementary Figure 3

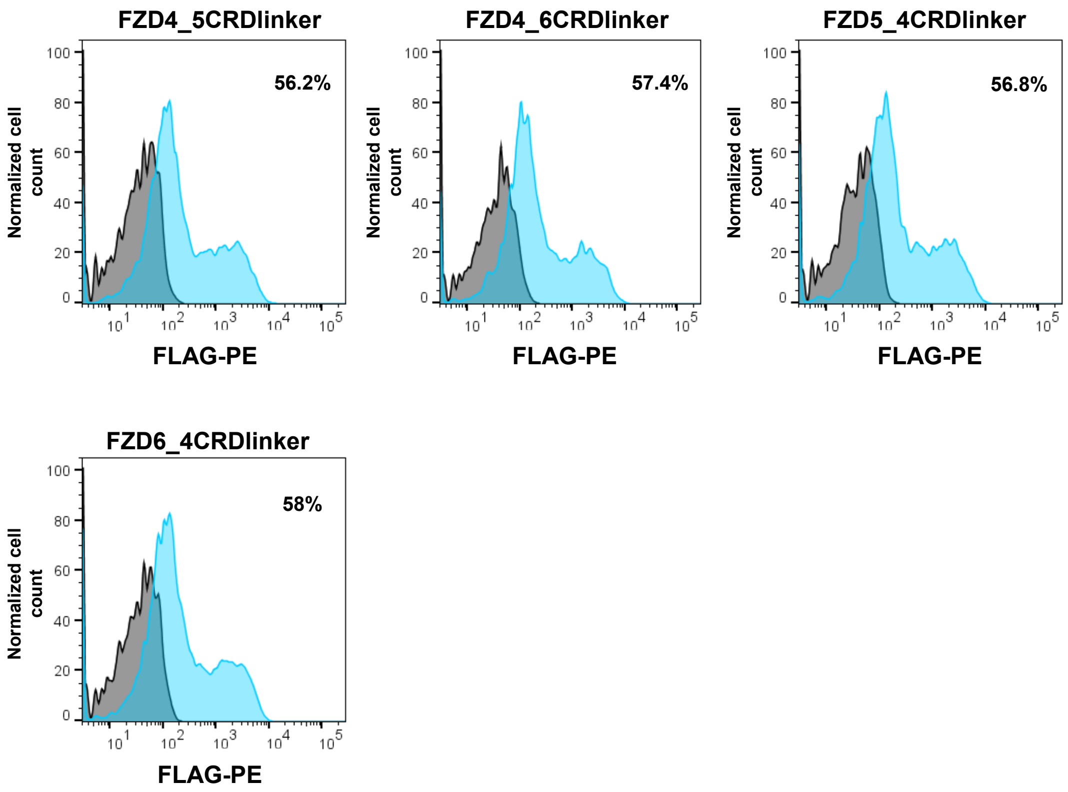

c

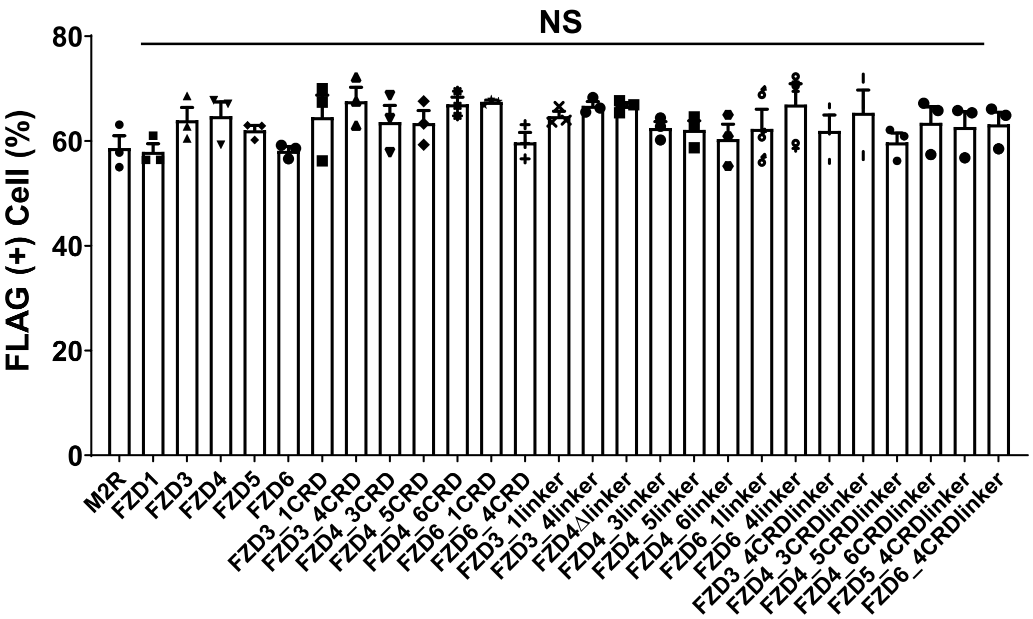

Supplementary Figure 3 Flow cytometry histograms of FZD expressing cells.

Surface expressed FZDs on cells were detected with PE-conjugated anti-FLAG antibody and quantified using FACS. **a** Parental gate of isotype and anti-FLAG antibody-stained group are drawn in SSC-A *vs.* FSC-A graph, excluding cell debris and dead cells. **b** Exemplar flow cytometry histograms of various FZD mutant constructs are shown, out of three replicates, overlayed with isotype control. **c** Percentage of cells expressing FZD calculated from flow cytometry histograms are plotted as a bar graph. The error bar indicates the SEM of n=three independent experiments. with error bars indicating SEM of three repeats. Statistical comparison was done with one-way ANOVA followed by Tukey's test. 'NS' represents not significant.

Supplementary Figure 4

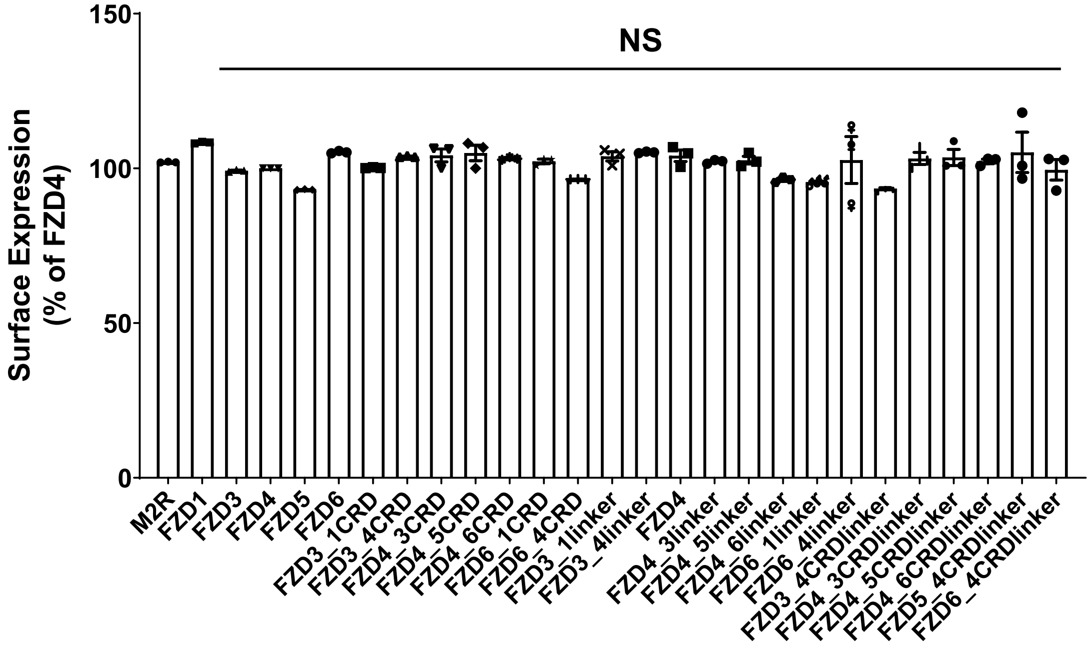

Supplementary Figure 4 Surface ELISA analysis of various FZD mutant constructs.

Surface expressed FZDs on cells were detected with anti-FLAG antibody. The expression level of each FZD construct was normalized with FZD4 wild-type as 100%. The results are plotted as bar graphs. The error bar indicates the SEM of n=three independent experiments. Statistical comparison was done with one-way ANOVA followed by the Tukey's test. 'NS' represents 'not significant'.

Supplementary Figure 5

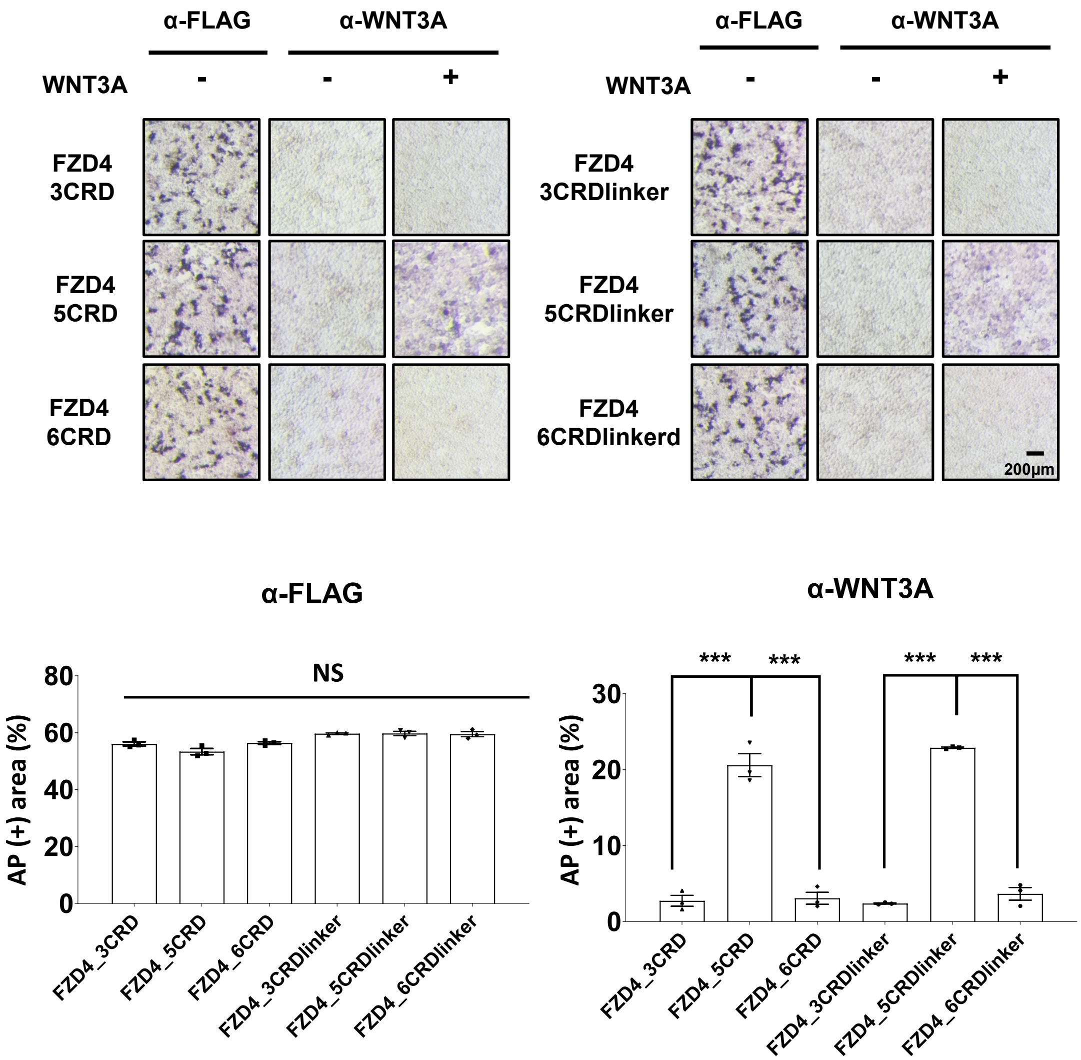

Supplementary Figure 5 AP assay of various FZD4 mutants to examine binding with WNT3A.

FZD by itself was stained via anti-FLAG antibody and FZD-bound WNT3A was stained via anti-WNT3A antibody. Antibodies bound to cells were visualized with NBT/BCIP substrates. AP-stained area was quantified using ImageJ and results are displayed as bar graphs. The error bar indicates the SD of n=three replicates experiments. Statistical comparisons were performed using one-way ANOVA followed by the Tukey's test. 'NS' represents 'not significant', and '***' represents P < 0.001. Scale bar indicates 200 μm.

Supplementary Figure 6

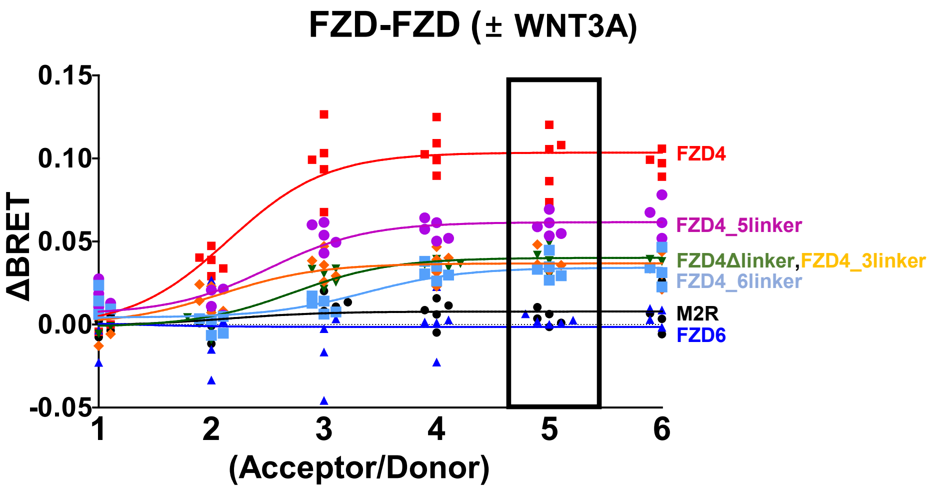

Supplementary Figure 6 Saturation BRET assay to assess FZD homo-oligomerization upon WNT3A treatment.

Saturation BRET assays were performed to find ideal ratio of BRET acceptor to donor. ΔBRET was calculated by subtracting vehicle-treated BRET ratio from WNT3A-treated BRET ratio for each pair. All BRET assays for FZD homo-oligomerization were performed with the acceptor-to-donor ratio marked with a black box. Data points show ΔBRET values from all five replicates and the graphs are fitted with nonlinear regression.

Supplementary Figure 7

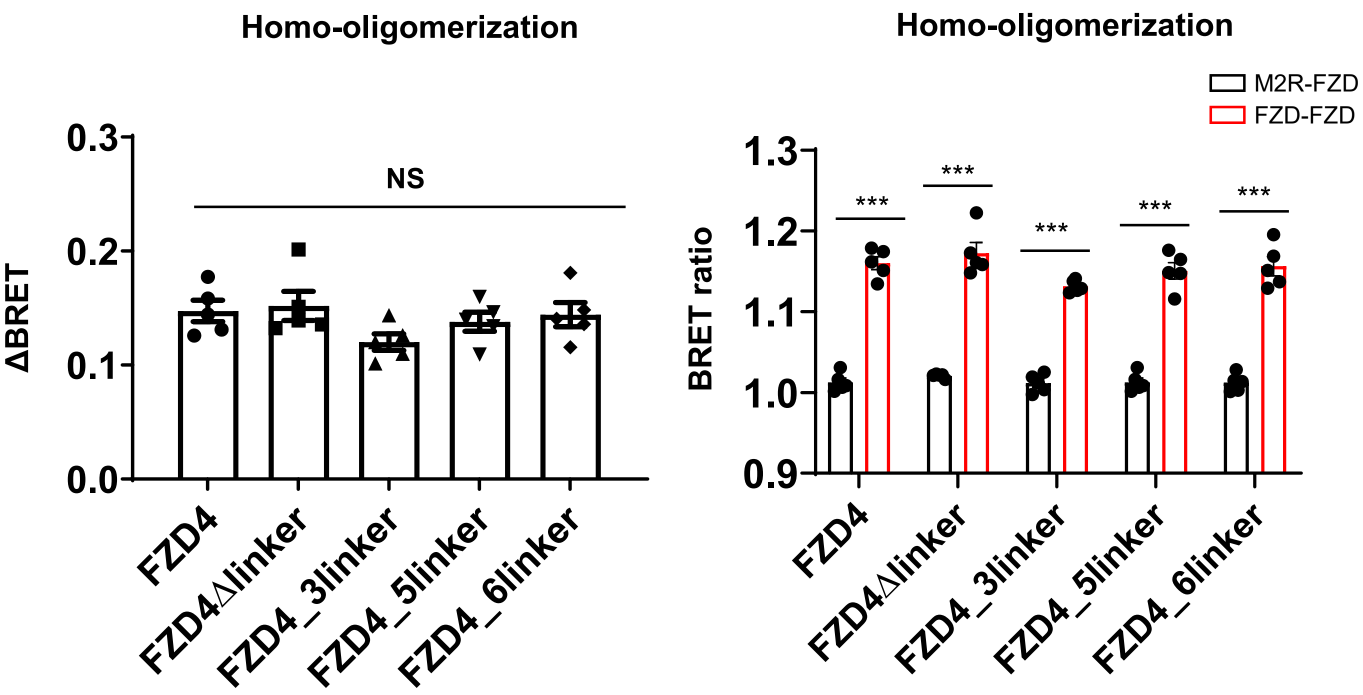

Supplementary Figure 7 BRET assay for FZD4 mutants to assess FZD homo-oligomerization at basal state.

ΔBRET and BRET ratios for receptor BRET pairs are plotted. Each BRET value indicates the homo-oligomerization level of each receptor pair in the absence of exogenous ligand treatment. A functionally unrelated M2R was used as a negative control and BRET ratio of M2R-FZD pair was subtracted from that of FZD-FZD pair to obtain ΔBRET. The error bar indicates the SEM of n=five independent experiments. Statistical comparisons were performed using one-way ANOVA followed by the Tukey's test and Two-tailed t test. 'NS' represents not significant and '***' means $P < 0.001$.

Supplementary Figure 8

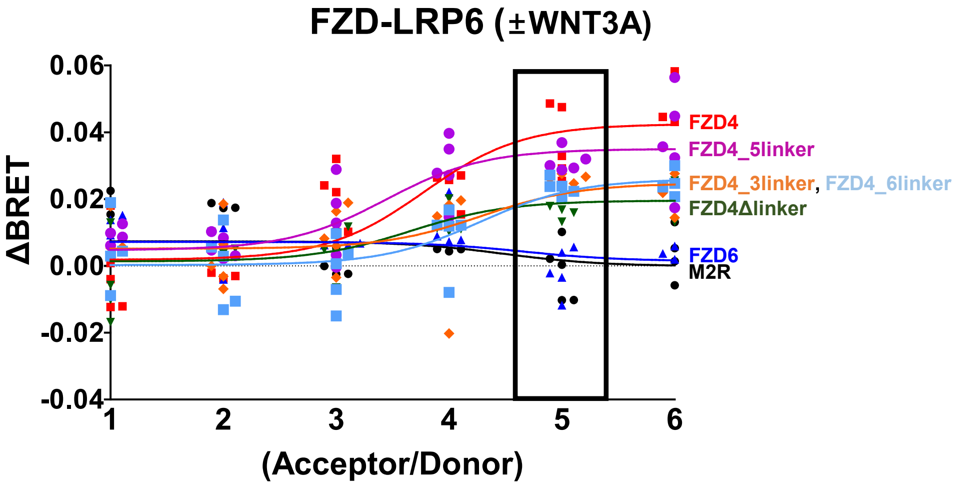

Supplementary Figure 8 Saturation BRET assay to assess LRP6 recruitment upon WNT3A treatment.

Saturation BRET assays were performed to find ideal ratio of BRET acceptor to donor. ΔBRET was calculated by subtracting vehicle-treated BRET ratio from WNT3A-treated BRET ratio for each pair. All BRET assays for LRP6 recruitment were performed with the acceptor-to-donor ratio marked with a black box. Data points show ΔBRET values from all five replicates and the graphs are fitted nonlinear regression.

Supplementary Figure 9

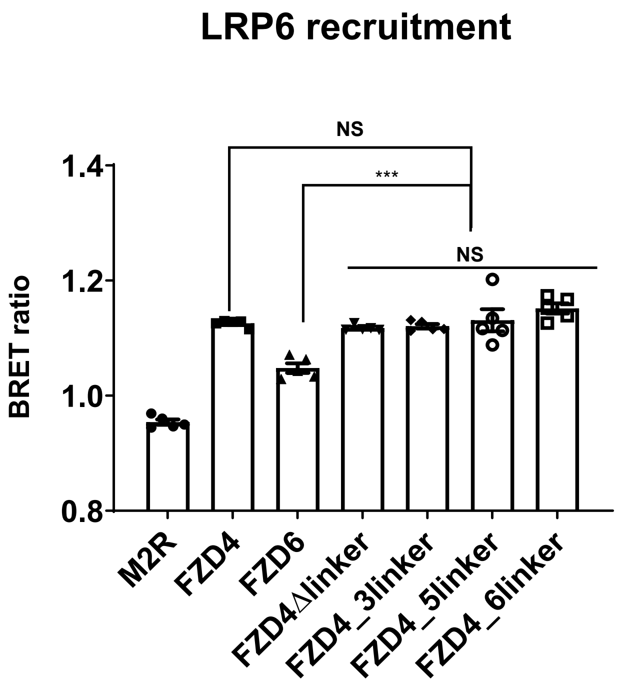

Supplementary Figure 9 BRET assay to assess LRP6 recruitment at basal state.

Basal BRET ratios for receptor and LRP6 pairs are plotted, indicating the degree of LRP6 recruitment to receptor in the absence of exogenous ligand treatment. A functionally unrelated M2R was included as a negative control. The error bars indicate the SEM of n=five independent experiments. Statistical comparisons were performed using one-way ANOVA followed by the Tukey's test. 'NS' represents not significant, and '***' represents $P < 0.001$.

Supplementary Figure 10

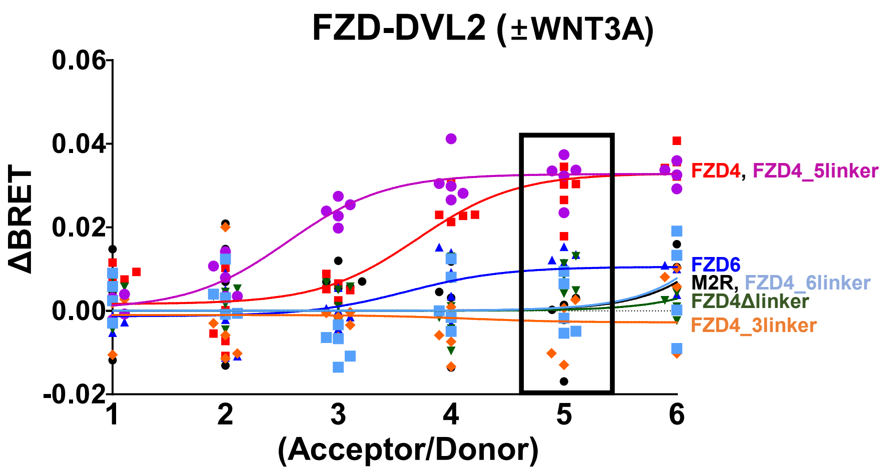

Supplementary Figure 10 Saturation BRET assay to assess DVL2 recruitment upon WNT3A treatment.

Saturation BRET assays were performed to find ideal ratio of BRET acceptor to donor. ∆BRET was calculated by subtracting vehicle-treated BRET ratio from WNT3A-treated BRET ratio for each pair. All BRET assays for DVL2 recruitment to receptor were performed with the acceptor-to-donor ratio marked with a black box. Data points show ∆BRET values from all five replicates and the graphs are fitted with nonlinear regression.

Supplementary Figure 11

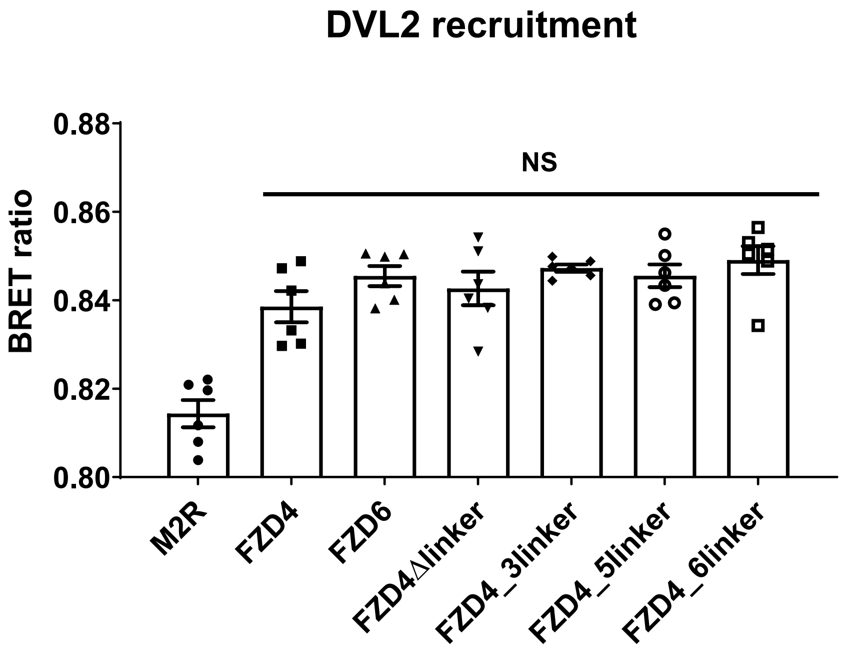

Supplementary Figure 11 BRET assay for FZD4 mutants to assess DVL2 recruitment at basal state.

BRET assay was performed with FZD-Rluc/DVL2-YFP pair to monitor DVL2 recruitment to receptor at basal state. M2R was included as a negative control. The error bars indicate the SEM of n=six independent experiments. Statistical comparison was done with one-way ANOVA followed by the Tukey's test. 'NS' represents not significant.

Supplementary Figure 12

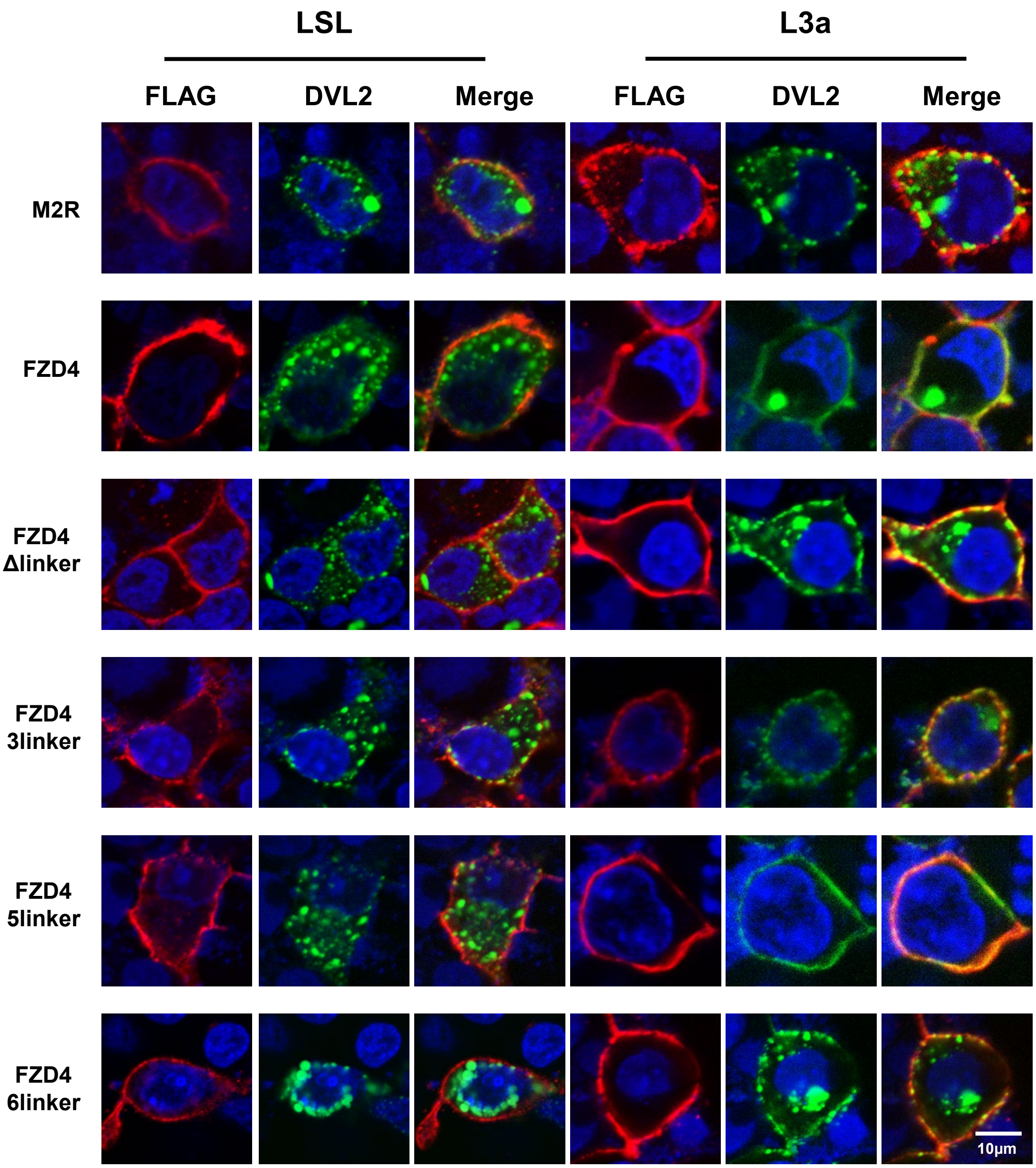

Supplementary Figure 12 High magnification immunofluorescence images showing DVL2 recruitment to FZD upon L3a serum treatment. Immunofluorescence images were observed with a confocal microscope. All receptors were visualized with anti-FLAG antibody (red) and nuclei were stained with Hoechst 33542 (blue). DVL2 was detected with its N-terminal YFP tag (green). Scale bar indicates 10 μm.

Supplementary Figure 13

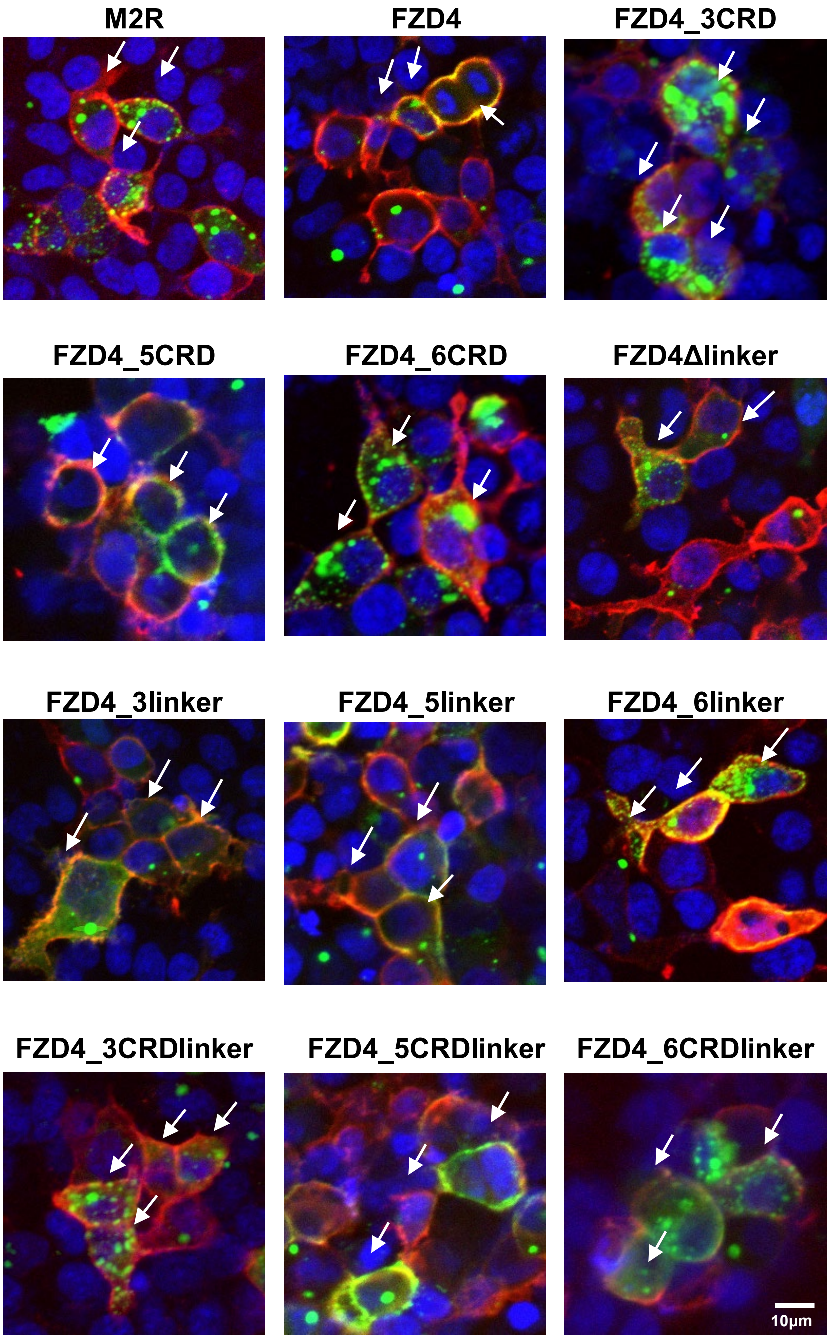

Supplementary Figure 13 Low magnification immunofluorescence images showing DVL2 recruitment to FZD upon L3a treatment. Immunofluorescence images were observed with a confocal microscope. All receptors were visualized with anti-FLAG antibody (red) and nuclei were stained with Hoechst 33542 (blue). DVL2 was detected with its N-terminal YFP tag (green). Scale bar indicates 10 μm.

Supplementary Figure 14

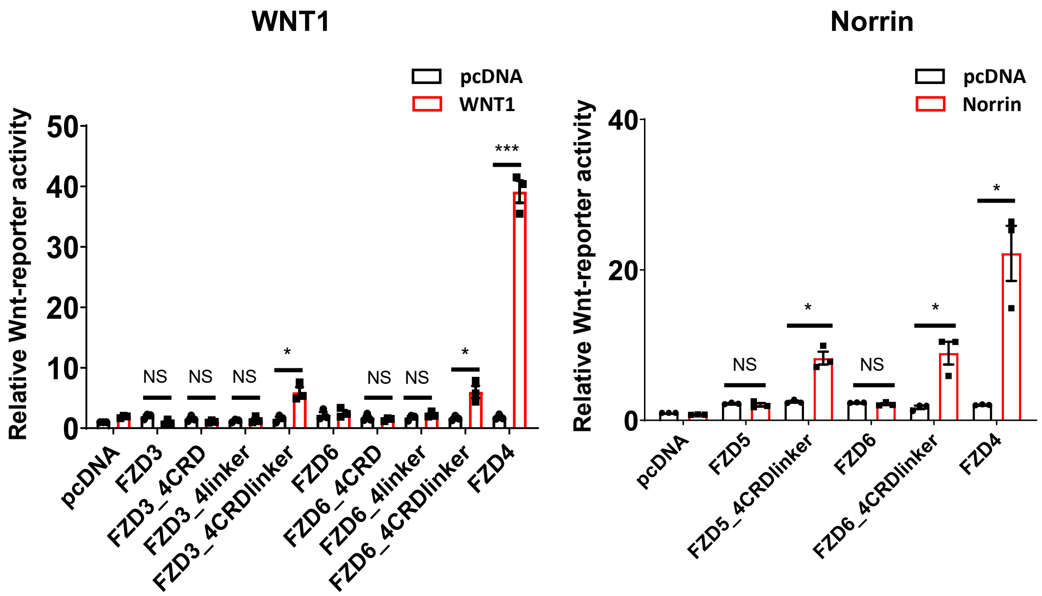

Supplementary Figure 14 TOPFlash assays of FZD3 and FZD6 mutant constructs with canonical ligands.

The effect of various FZD3 and FZD6 mutants on WNT1 or Norrin signaling is observed through TOPFlash assays. The error bar indicates the SEM of n=three independent experiments. Statistical comparisons were performed using Two tailed t-test ‘NS’ means not significant, ‘***’ means P < 0.001, and ‘*’ means P <0.05.

Supplementary Figure 15

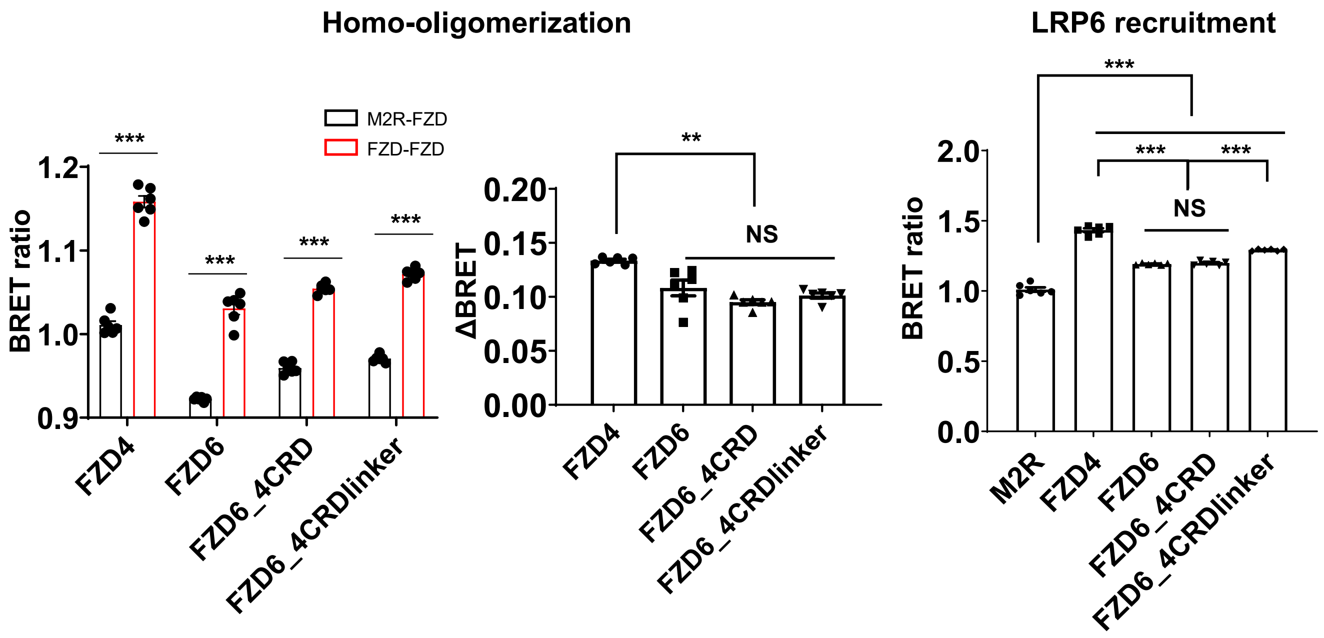

Supplementary Figure 15 Homo-oligomerization and LRP6 recruitment of FZD6 CRD and CRDlinker swapped mutants at basal state.

Basal BRET ratios for FZD6 CRD/CRDlinker mutants are plotted, one for homo-oligomerization and the other for LRP6 recruitment as indicated. M2R was included as a negative control. BRET ratio of M2R-FZD pair was subtracted from that of FZD-FZD pair to obtain ΔBRET for homo-oligomerization. The error bar indicates the SEM of n=six independent experiments. Statistical comparisons were performed using one-way ANOVA followed by Tukey's test and Two-tailed t-test. 'NS' means not significant, '***' means $P < 0.001$, and '**' means $P < 0.01$.

Supplementary Figure 16

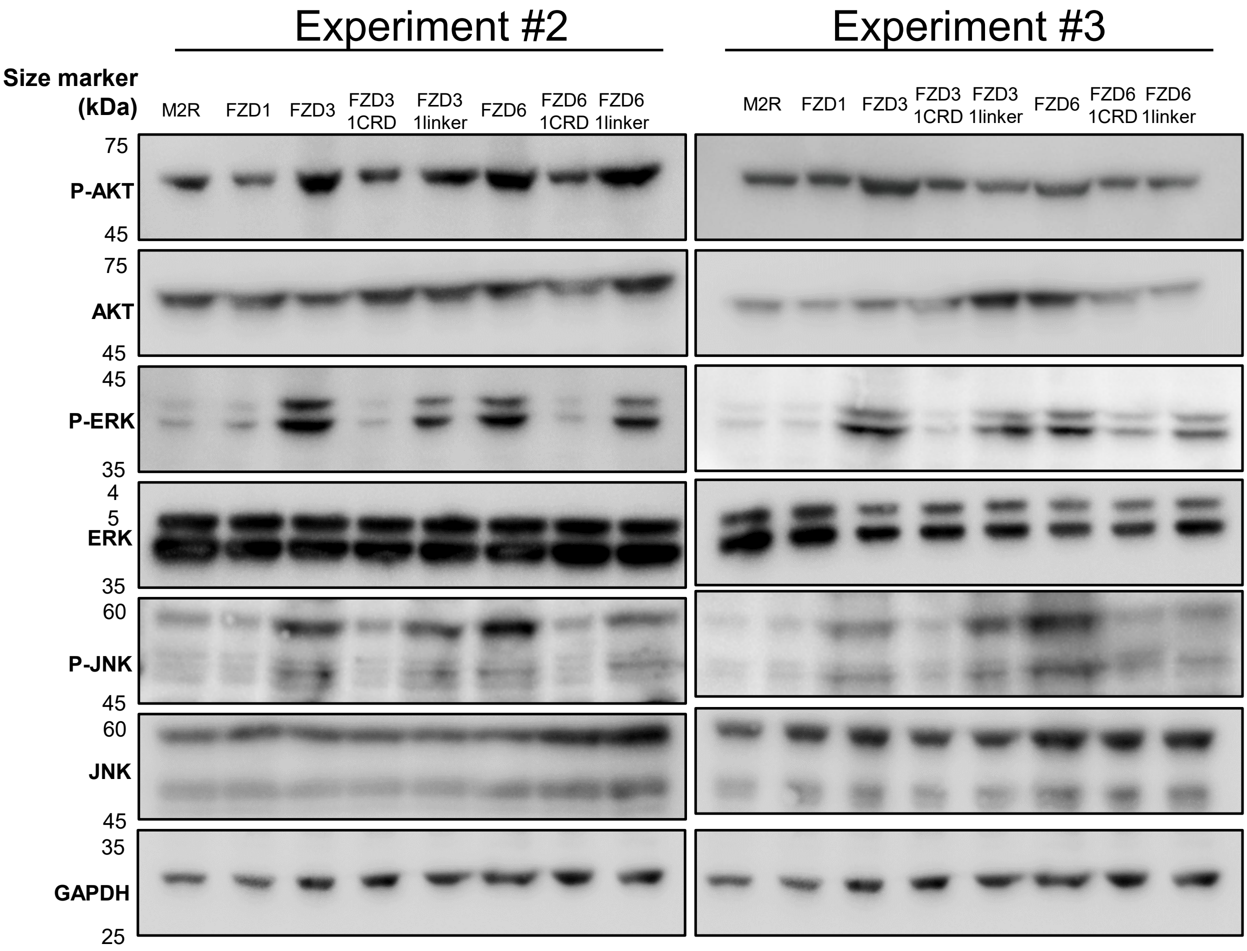

Supplementary Figure 16 Western blot analysis of the phosphorylation levels of various signaling molecules.

Western blot images of the phosphorylation levels of ERK, AKT and JNK in response to WNT5A in dFZD1-10 $^{-/-}$ cells using FZD3 and FZD6 related chimeric constructs. Uncropped western blot images are shown in Source data. Three independent western blots were quantitatively analyzed as shown in Figure 6a.

Supplementary Figure 17

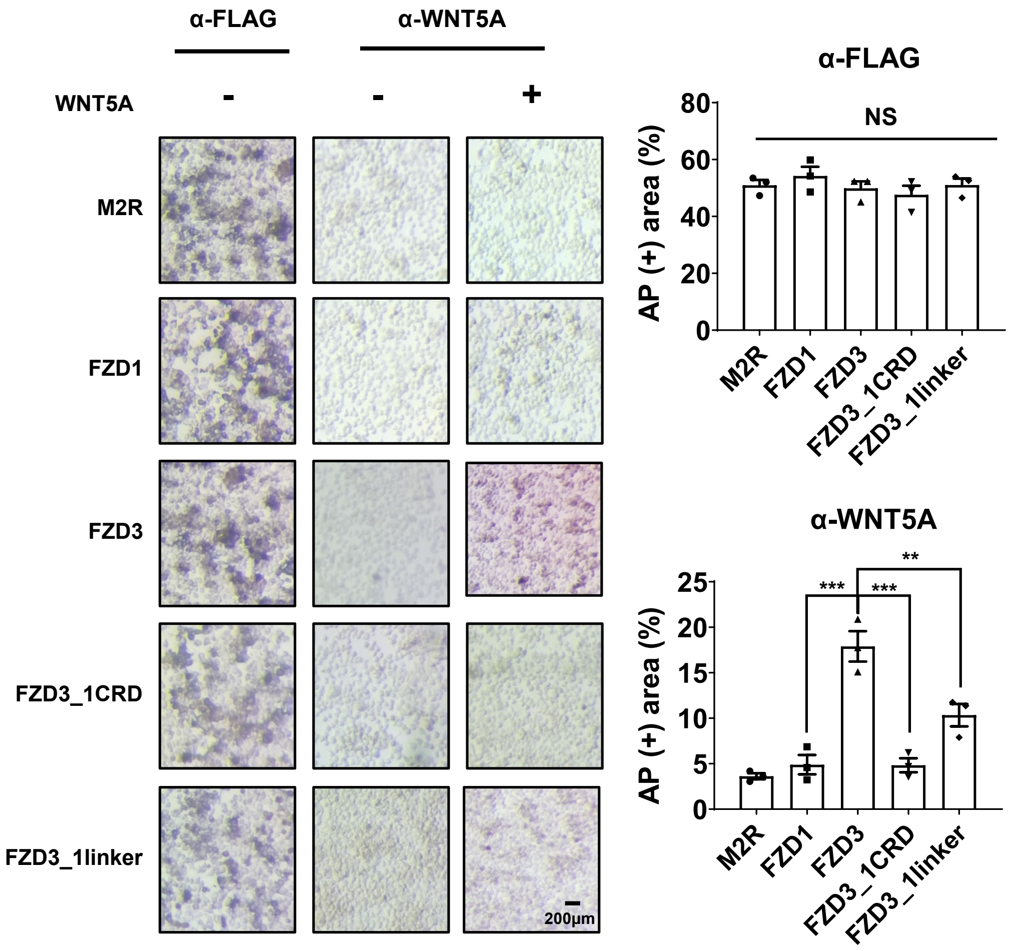

Supplementary Figure 17 AP staining assays of FZD3 mutants.

AP-stained images of transfected dFZD1-10 $^{-/-}$ cells with the FZD3 CRD and linker swapped mutants, with or without L5a treatment. Each graph represents AP-stained area of surface expressed FLAG and receptor bound WNT5A, respectively. The error bar indicates the SD of n=three replicates experiments. Statistical comparisons were performed using one-way ANOVA followed by Tukey's test. 'NS' means not significant, '***' means P < 0.001, and '**' means P <0.01. Scale bar indicates 200 μm.

Supplementary Figure 18

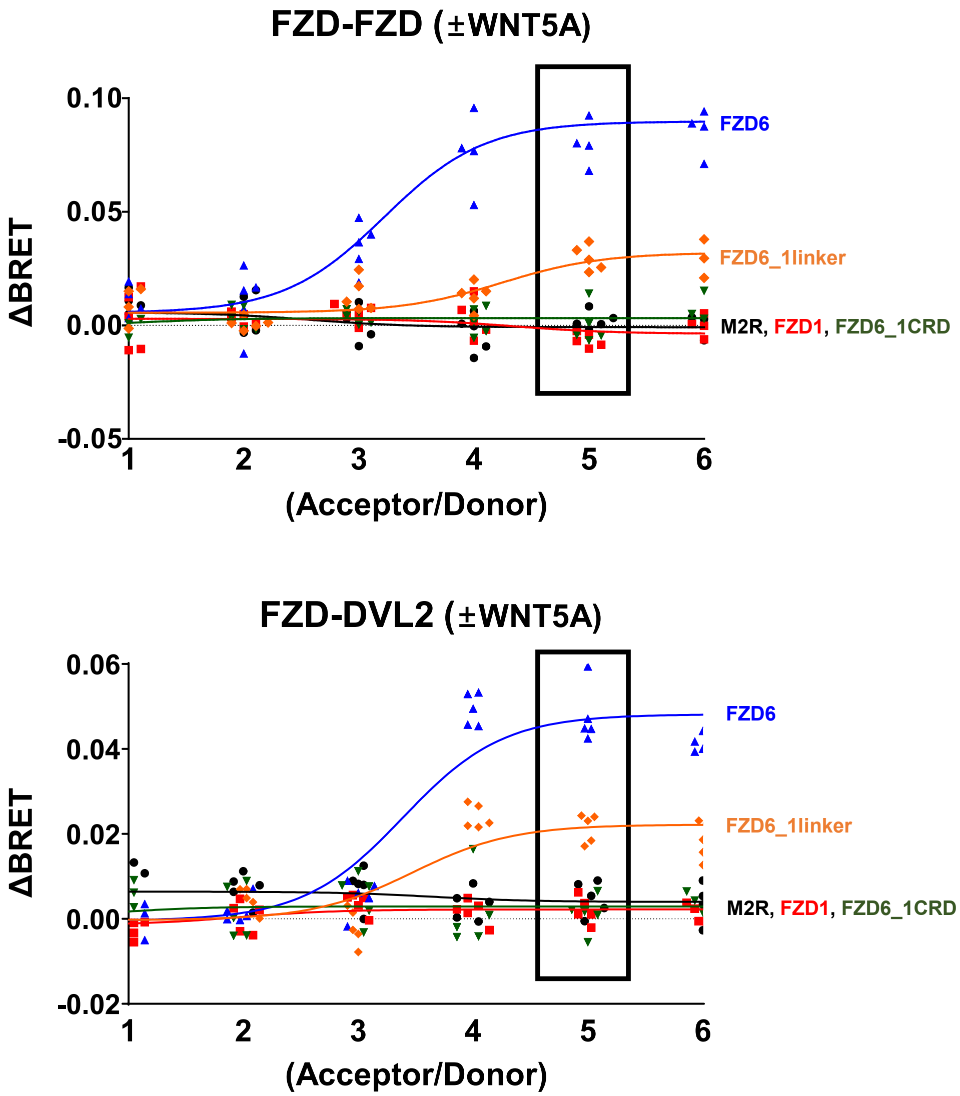

Supplementary Figure 18 Saturation BRET assays for FZD6 mutants to assess FZD homo-oligomerization and DVL2 recruitment.

Saturation BRET assays were performed to find ideal ratio of BRET acceptor to donor. ΔBRET was calculated by subtracting vehicle-treated BRET ratio from WNT5A-treated BRET ratio for each pair. All BRET assays for FZD homo-oligomerization and DVL2 recruitment were performed with the acceptor-to-donor ratio marked with each black box. Data points show ΔBRET values from all five replicates and the graphs are fitted with nonlinear regression.

Supplementary Figure 19

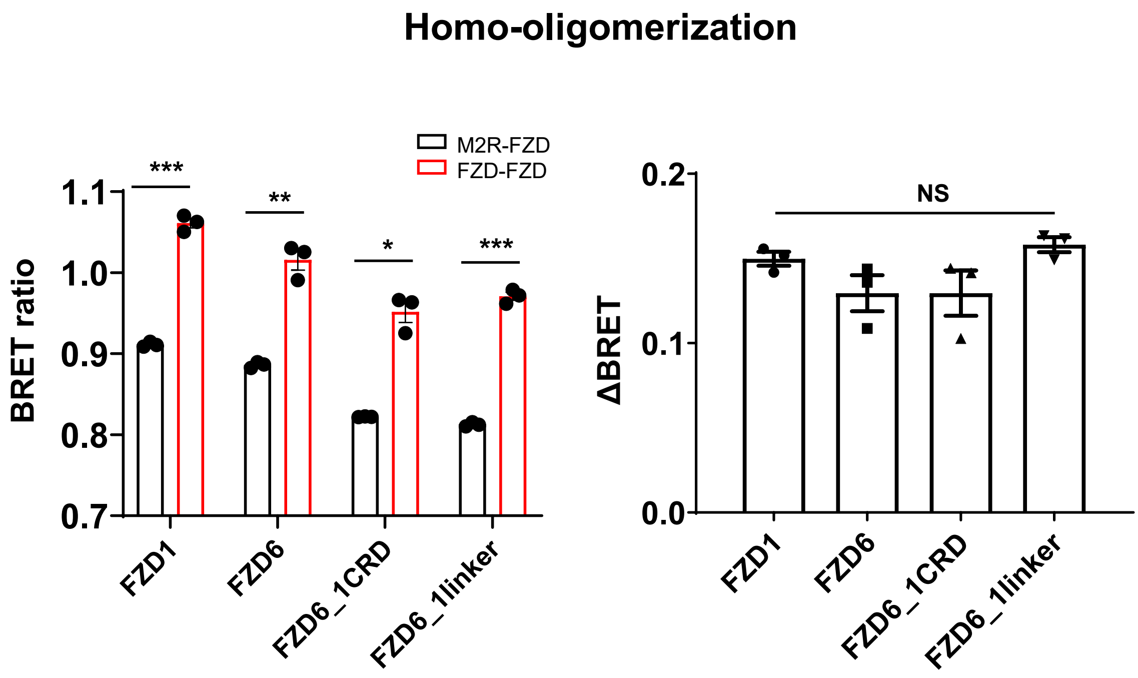

Supplementary Figure 19 BRET assay for FZD6 mutants to assess homo-oligomerization at basal state.

ΔBRET and BRET ratios for receptor BRET pairs are plotted. Each BRET value indicates the homo-oligomerization level of each receptor pair in the absence of exogenous ligand treatment. A functionally unrelated M2R was used as a negative control and BRET ratio of M2R-FZD pair was subtracted from that of each FZD-FZD pair to obtain ΔBRET. The error bars indicate the SEM of n=three independent experiments. Statistical comparisons were performed using one-way ANOVA followed by Tukey's test and Two-tailed t test. 'NS' means not significant, '***' means P < 0.001, '**' means P < 0.01 and '*' means P < 0.05.

Supplementary Figure 20

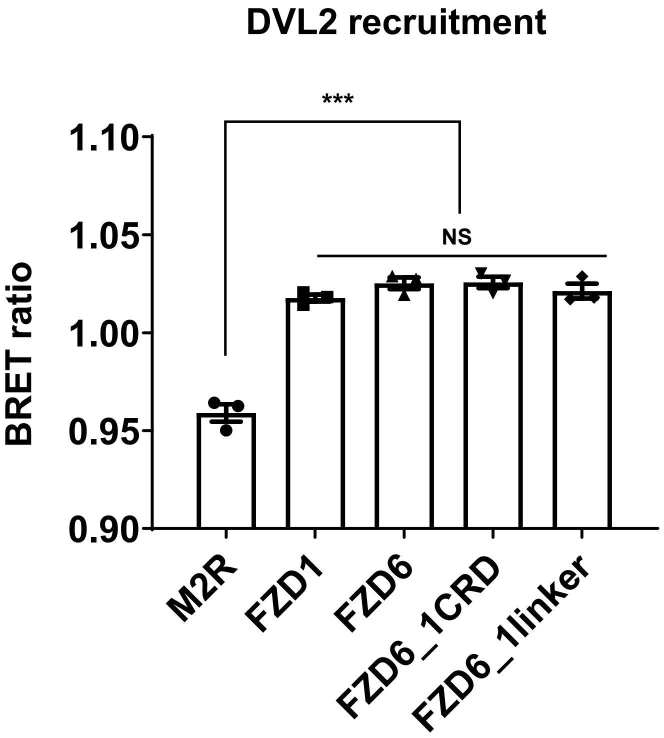

Supplementary Figure 20 BRET assay for FZD6 mutants to assess DVL2 recruitment at basal state.

BRET assay was performed with FZD-Rluc/DVL2-YFP pair to monitor DVL2 recruitment to receptor in the absence of exogenous ligand treatment. A functionally unrelated M2R was used as a negative control. The error bars indicate the SEM of n=three independent experiments. Statistical comparisons were performed using one-way ANOVA followed by Tukey's test. 'NS' means not significant, and '***' means P < 0.001.

Supplementary Figure 21

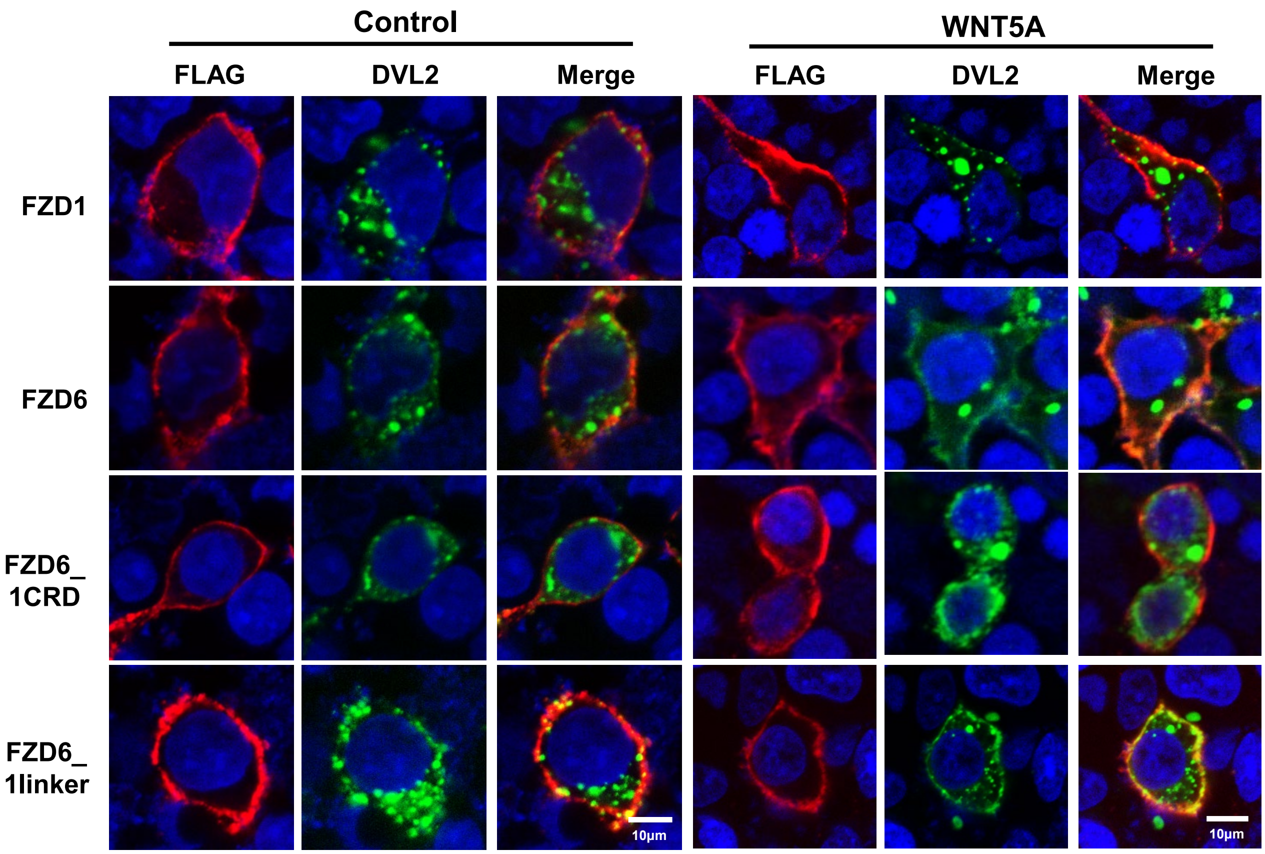

Supplementary Figure 21 High magnification immunofluorescence images showing DVL2 recruitment to FZD upon WNT5A treatment. Red indicates FZDs detected with anti-FLAG antibody and blue indicates Hoechst 33542 stained nuclei. DVL2 was detected with its N-terminal YFP tag, shown in green. Scale bar indicates 10 μm.

Supplementary Figure 22

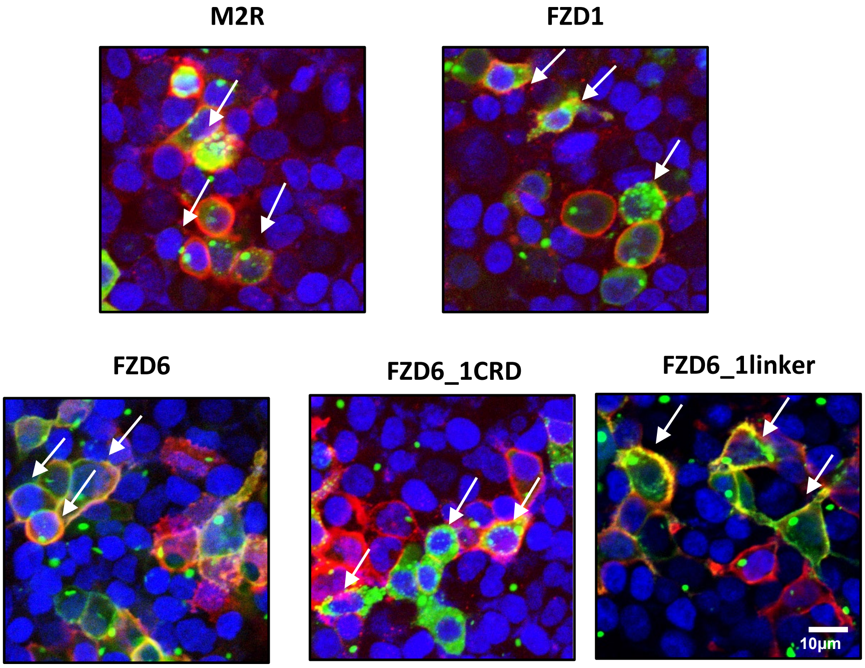

Supplementary Figure 22 Low magnification immunofluorescence images showing DVL2 recruitment to FZD upon WNT5A treatment. Red indicates FZDs detected with anti-FLAG antibody and blue indicates Hoechst 33542 stained nuclei. DVL2 was detected with its N-terminal YFP tag, shown in green. Scale bar indicates 10 μm.

Supplementary Figure 23

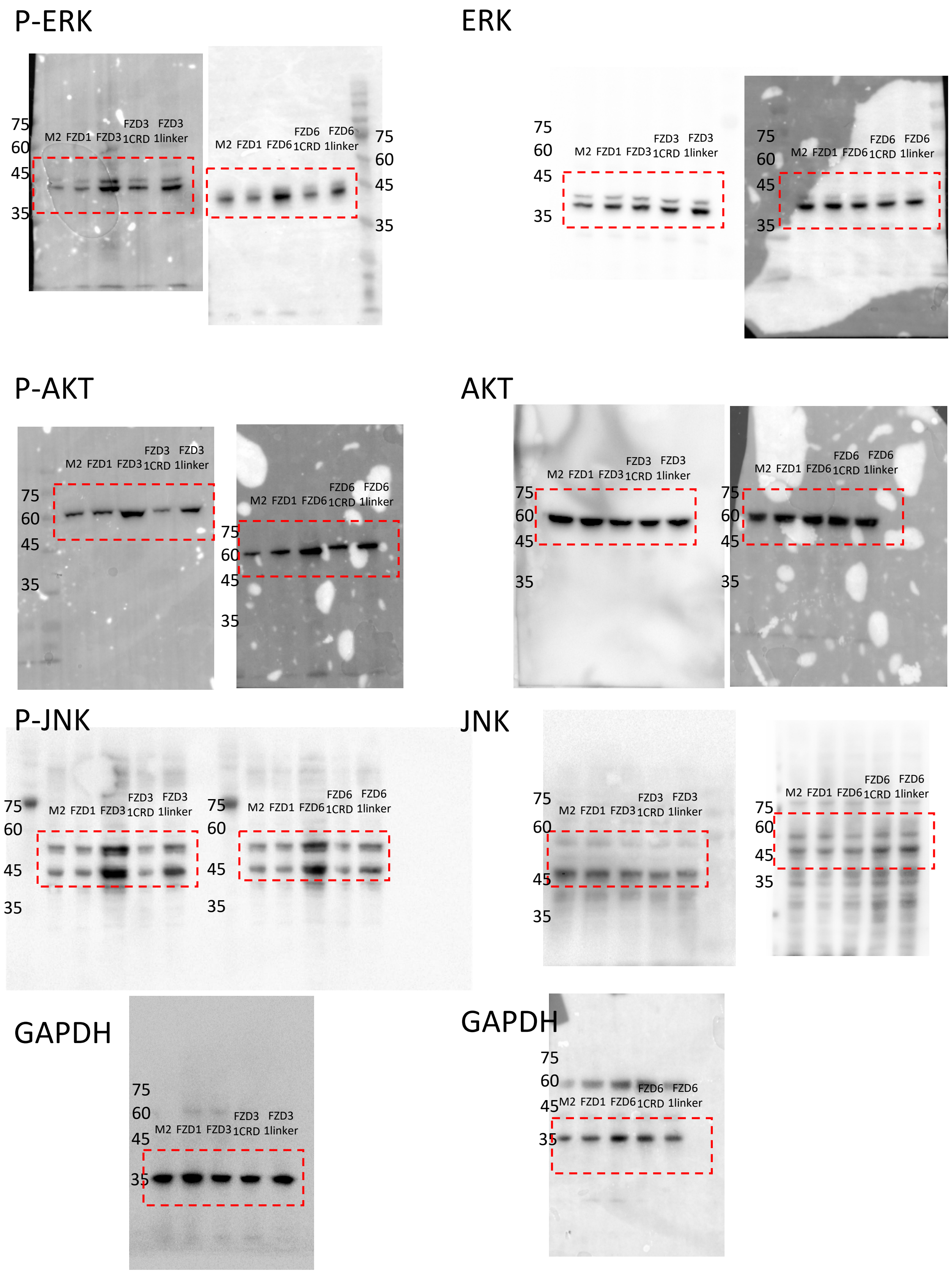

Supplementary Figure 23 Uncropped Western blot images of Fig. 6a.

Supplementary Table 1. Construct information of FZD mutants

Category	Mutant name	Description
CRD mutants	**FZD3_1CRD**	FLAG-FZD1 (70-229)-FZD3 (136-666)
	**FZD3_4CRD**	FLAG-FZD4 (41-160)-FZD3 (136-666)
	**FZD4_3CRD**	FLAG-FZD3 (23-135)-FZD4 (161-537)
	**FZD4_5CRD**	FLAG-FZD5 (27-149)-FZD4 (161-537)
	**FZD4_6CRD**	FLAG-FZD6 (19-131)-FZD4 (161-537)
	**FZD5_4CRD**	FLAG-FZD4 (41-160)-FZD5 (150-585)
	**FZD6_1CRD**	FLAG-FZD1 (70-229)-FZD6 (132-706)
	**FZD6_4CRD**	FLAG-FZD4 (41-160)-FZD6 (132-706)
Linker mutants	**FZD3_1linker**	FLAG-FZD3 (23-135)-FZD1 (230-299)-FZD3 (189-666)
	**FZD3_4linker**	FLAG-FZD3 (23-135)-FZD4 (161-203)-FZD3 (189-666)
	**FZD4Δlinker**	FLAG-FZD4 (41-160)-GGSGG-FZD4 (204-537)
	**FZD4_3linker**	FLAG-FZD4 (41-160)-FZD3 (136-188)-FZD4 (204-537)
	**FZD4_5linker**	FLAG-FZD4 (41-160)-FZD5 (150-221)-FZD4 (204-537)
	**FZD4_6linker**	FLAG-FZD4 (41-160)-FZD6 (132-184)-FZD4 (204-537)
	**FZD6_1linker**	FLAG-FZD6 (19-131)-FZD1 (230-299)-FZD6 (185-706)
	**FZD6_4linker**	FLAG-FZD6 (19-131)-FZD4 (161-203)-FZD6 (185-706)
CRDlinker mutants	**FZD3_4CRDlinker**	FLAG-FZD4 (41-203)-FZD3 (189-666)
	**FZD4_3CRDlinker**	FLAG-FZD3 (23-188)-FZD4 (204-537)
	**FZD4_5CRDlinker**	FLAG-FZD5 (27-221)-FZD4 (204-537)
	**FZD4_6CRDlinker**	FLAG-FZD6 (19-184)-FZD4 (204-537)
	**FZD5_4CRDlinker**	FLAG-FZD4 (41-203)-FZD5 (222-585)
	**FZD6_4CRDlinker**	FLAG-FZD4 (41-203)-FZD6 (185-706)
