# Supplementary material for: Functional role of the Frizzled linker domain in the Wnt signaling pathway
Source: Commun Biol. 2022 May 5;5:421. doi: 10.1038/s42003-022-03370-4 (PMC9072438; doi:10.1038/s42003-022-03370-4)
Supplement: Supplementary file 3 — Description of Additional Supplementary Files [file 42003_2022_3370_MOESM3_ESM.pdf]

Description of Additional Supplementary Files

File name: Supplementary Data 1
Description: Source data for figures.

File name: Supplementary Data 2
Description: List of PCR primers.
