# Supplementary material for: Functional role of the Frizzled linker domain in the Wnt signaling pathway
Source: Commun Biol. 2022 May 5;5:421. doi: 10.1038/s42003-022-03370-4 (PMC9072438; doi:10.1038/s42003-022-03370-4)
Supplement: Supplementary file 6 — Reporting Summary [file 42003_2022_3370_MOESM6_ESM.pdf]

nature portfolio

Corresponding author(s): Hee-Jung Choi

Last updated by author(s): Mar 23, 2022

# Reporting Summary

Nature Portfolio wishes to improve the reproducibility of the work that we publish. This form provides structure for consistency and transparency in reporting. For further information on Nature Portfolio policies, see our Editorial Policies and the Editorial Policy Checklist.

## Statistics

For all statistical analyses, confirm that the following items are present in the figure legend, table legend, main text, or Methods section.

| n/a | Confirmed | |
|---|---|---|
| ☐ | ☒ | The exact sample size (*n*) for each experimental group/condition, given as a discrete number and unit of measurement |
| ☐ | ☒ | A statement on whether measurements were taken from distinct samples or whether the same sample was measured repeatedly |
| ☐ | ☒ | The statistical test(s) used AND whether they are one- or two-sided<br>*Only common tests should be described solely by name; describe more complex techniques in the Methods section.* |
| ☒ | ☐ | A description of all covariates tested |
| ☐ | ☒ | A description of any assumptions or corrections, such as tests of normality and adjustment for multiple comparisons |
| ☒ | ☐ | A full description of the statistical parameters including central tendency (e.g. means) or other basic estimates (e.g. regression coefficient) AND variation (e.g. standard deviation) or associated estimates of uncertainty (e.g. confidence intervals) |
| ☐ | ☒ | For null hypothesis testing, the test statistic (e.g. *F*, *t*, *r*) with confidence intervals, effect sizes, degrees of freedom and *P* value noted<br>*Give P values as exact values whenever suitable.* |
| ☒ | ☐ | For Bayesian analysis, information on the choice of priors and Markov chain Monte Carlo settings |
| ☒ | ☐ | For hierarchical and complex designs, identification of the appropriate level for tests and full reporting of outcomes |
| ☒ | ☐ | Estimates of effect sizes (e.g. Cohen's *d*, Pearson's *r*), indicating how they were calculated |

*Our web collection on statistics for biologists contains articles on many of the points above.*

## Software and code

Policy information about availability of computer code

| | |
|---|---|
| Data collection | Fluorescence images: Zeiss Zen blue edition (Ver3.3)<br>Flow cytometry:  FACSDiva 6.13 |
| Data analysis | General statistical analysis for GraphPad Prism 8 and Microsoft Excel<br>Western blot, AP-stained assay quantification for Image J<br>Flow cytometry: Flowjo_V10 |

For manuscripts utilizing custom algorithms or software that are central to the research but not yet described in published literature, software must be made available to editors and reviewers. We strongly encourage code deposition in a community repository (e.g. GitHub). See the Nature Portfolio guidelines for submitting code & software for further information.

## Data

Policy information about availability of data

All manuscripts must include a data availability statement. This statement should provide the following information, where applicable:

- Accession codes, unique identifiers, or web links for publicly available datasets
- A description of any restrictions on data availability
- For clinical datasets or third party data, please ensure that the statement adheres to our policy

All of the data supporting the findings of this study are stated in this article and in the Supplementary information.

# Field-specific reporting

Please select the one below that is the best fit for your research. If you are not sure, read the appropriate sections before making your selection.

☒ Life sciences ☐ Behavioural & social sciences ☐ Ecological, evolutionary & environmental sciences

For a reference copy of the document with all sections, see nature.com/documents/nr-reporting-summary-flat.pdf

# Life sciences study design

All studies must disclose on these points even when the disclosure is negative.

| | |
|---|---|
| Sample size | No sample size was calculated |
| Data exclusions | No data was excluded from the analysis |
| Replication | Three independent experiments on TOPFlash assay (Fig 2 a,b ,Fig 5 a,b and Supplementary Fig 14), three independent experiments on ELISA based surface expression assay (Supplementary Fig 4), three independent experiments on BRET assay (Fig 4 a,b,c ,Fig 6 c,d, Supplementary Fig 19 and Supplementary Fig 20), five independent experiments on BRET assay (Supplementary Fig 7 and Supplementary Fig 9), six independent experiments on BRET assay (Fig 5d,e, Supplementary Fig 11, Supplementary Fig 15), five fields of view were randomly chosen in each confocal dish using DAPI channel to avoid bias in cell selection (Supplementary Fig 13 and Supplementary Fig 22), three fields of view were randomly chosen in each confocal dish using DAPI channel to avoid bias in cell selection (Supplementary Fig 12 and Supplementary Fig 21), three fields of view were randomly chosen in each confocal dish using AP stained area to avoid bias in cell section (Fig 3, Fig 5c, Fig 6b, Supplementary Fig 5 and Supplementary Fig 17), five replicates on Saturation BRET assay (Supplementary Fig 6, 8, 10 and 18), Three independent experiments on Western blot (Fig 6a and Supplementary Fig 16) |
| Randomization | We randomly took fluorescence microscopic images and AP stained images. |
| Blinding | The researchers were not blinded for this study due to feasibility of experiments. |

# Reporting for specific materials, systems and methods

We require information from authors about some types of materials, experimental systems and methods used in many studies. Here, indicate whether each material, system or method listed is relevant to your study. If you are not sure if a list item applies to your research, read the appropriate section before selecting a response.

## Materials & experimental systems

| n/a | Involved in the study |
|---|---|
| ☐ | ☒ Antibodies |
| ☐ | ☒ Eukaryotic cell lines |
| ☒ | ☐ Palaeontology and archaeology |
| ☒ | ☐ Animals and other organisms |
| ☒ | ☐ Human research participants |
| ☒ | ☐ Clinical data |
| ☒ | ☐ Dual use research of concern |

## Methods

| n/a | Involved in the study |
|---|---|
| ☒ | ☐ ChIP-seq |
| ☐ | ☒ Flow cytometry |
| ☒ | ☐ MRI-based neuroimaging |

## Antibodies

| | |
|---|---|
| Antibodies used | Anti-DYKDDDDK rabbit mAb (1:1000, Cell Signaling Technology, 14793), PE conjugated Anti-DYKDDDDK rabbit mAb (1:100, Cell Signaling Technology,98533), Rabbit PE conjugated isotype control mAb (1:100, Cell Signaling Technology, 5742), Donkey anti-rabbit secondary antibody Alexa Fluor 555 (1:1000, Thermofisher scientific, A-31572), Goat anti-rabbit secondary antibody Alexa Fluor 488 (1:1000, Thermofisher scientific, A-11034), WNT3A polyclonal antibody unconjugated (1:1000, Thermofisher scientific, PA5-44946), WNT5A mAb unconjugated (1:1000 Thermofisher scientific MA-15502), Anti-rabbit IgG, AP linked antibody (1:1000, Cell Signaling Technology, 7054s), Anti-mouse IgG, AP linked antibody (1:1000, Cell Signaling Technology, 7056), p44/42 MAPK (Erk1/2) Rabbit mAb HRP conjugate (1:1000, Cell Signaling Technology, 4348), Phospho-p44/42 MAPK Rabbit mAb HRP conjugate (1:1000, Cell Signaling Technology, 8544), SAPK/JNK antibody rabbit (1:1000, Cell Signaling Technology, 9252), Phospho-SAPK/JNK rabbit mAb (1:1000, Thermofisher scientific, 44-682G), AKT (pan) Rabbit mAb (1:1000, Cell Signaling Technology, 4691), Phospho AKT rabbit mAb (1:1000, Cell Signaling Technology, 4060s), Anti-GAPDH HRP conjugate antibody (1:1000, Santa Cruz, sc-47724 HRP) |
| Validation | All antibodies are commercially available. No additional validation |

# Eukaryotic cell lines

Policy information about cell lines

| | |
|---|---|
| Cell line source(s) | dFZD1-10-/-cell line was gifted by Dr. Vanhollebeke (Eubelen, M. et al., Science., 2018) , LWnt-3A cell line (ATCC, CRL-2647™), LWnt5-A cell line (ATCC, CRL-2814™) |
| Authentication | None of cell lines have been authenticated. |
| Mycoplasma contamination | All cell lines were not tested for Mycoplasma contamination. |
| Commonly misidentified lines (See ICLAC register) | No commonly misidentified cell lines were used. |

# Flow Cytometry

## Plots

Confirm that:

☒ The axis labels state the marker and fluorochrome used (e.g. CD4-FITC).

☒ The axis scales are clearly visible. Include numbers along axes only for bottom left plot of group (a 'group' is an analysis of identical markers).

☒ All plots are contour plots with outliers or pseudocolor plots.

☒ A numerical value for number of cells or percentage (with statistics) is provided.

## Methodology

| | |
|---|---|
| Sample preparation | For quantification of the surface-expressed receptor, each FZD mutant construct was transfected into dFZD1-10-/- cells. After transfection, the samples were detached using 20 mM EDTA in PBS and then resuspended with flow cytometry buffer (1% FBS + 0.1% BSA in PBS). The cells were incubated with PE-conjugated either anti-FLAG antibody or isotype control antibody (Cell Signaling Technology, USA) in flow cytometry buffer and incubated for 30 min at 4° C. The stained cells were then washed and resuspended with the same buffer. |
| Instrument | FACS canto |
| Software | FACSDiva 6.13 |
| Cell population abundance | Sorting was not performed in this study |
| Gating strategy | Gate was set based on side scattering (SSC) and forward scattering (FSC) to exclude cell debris and dead cells. The cell count of the anti-FLAG antibody-stained sample and that of the isotype-stained sample were overlaid, and the area of the anti-FLAG stained sample that does not overlap with that of the isotype stained sample was taken as the target protein expressing percentage of the cells. |

☒ Tick this box to confirm that a figure exemplifying the gating strategy is provided in the Supplementary Information.
